# Views of “being vulnerable” among people attended by a Portuguese community association: a qualitative study

**DOI:** 10.1192/j.eurpsy.2023.2014

**Published:** 2023-07-19

**Authors:** C. Laranjeira, L. Cordeiro, A. Querido

**Affiliations:** 1 School of Health Sciences; 2 ciTechCare, Polytechnic of Leiria; 3 InPulsar, Leiria, Portugal

## Abstract

**Introduction:**

The concept of social vulnerability arises in the individual-collective relationship and points to changing social conditions, built on the basis of power relations. In this context, vulnerability can be directly related to the deterioration of social and civil rights, resulting in the weakening of individuals’ citizenship. On the other hand, vulnerability can also lead to the deterioration of mental health. Stigma and discrimination generate low self-esteem, decreased self-confidence, reduced motivation, and less hope for the future.

**Objectives:**

This study aimed to explore the perceptions and experiences of vulnerability from the perspective of vulnerable people and identify strategies they used to reduce vulnerability.

**Methods:**

This study employed a qualitative descriptive design. Using a purposeful sampling method, data were collected in April 2022. The following criteria were applied in the selection process: (1) adults with a personal sense of vulnerability or the experience of being vulnerable; and (2) understanding the Portuguese language and having reflective capacity.

**Results:**

A total of 12 respondents (6 male; 6 women) participated in study, mostly of middle-age. The manifestations of vulnerability reported by participants included being homeless, being a migrant, having an infectious disease, being drug dependent, experiencing a process of loss and grief and living socioeconomic difficulties. The data was summarized in terms of three major themes: (1) Conceptions about vulnerability, (2) Barriers imposed by vulnerability, and (3) Strategies for dealing with vulnerability. “Three subthemes were identified within the first theme: ontology condition that spreads, being alone “without network” and being exposed to external pressure (others). In the second theme, there were also three subthemes: discrimination/stigma, difficulties in social reintegration, and “my condition is difficult”. Lastly, in the third theme, we found four subthemes: the ability to ask for help/seek support, motivation, and commitment to behavioural change, not exposing others to the same risks, and ignoring the disapproving look of others” (Laranjeira et al., 2022, p.5).

**Conclusions:**

Our findings demonstrated that vulnerability is a dynamic process of being exposed to circumstances that influence individual outcomes. However, there is a conceptual gap: vulnerability is regarded negatively, but vulnerability also has the capacity to shift life priorities for the better.

**References:**

Laranjeira C., Piaça I., Vinagre H., Vaz AR., Ferreira S., Cordeiro L., Querido A. (2022). Vulnerability through the Eyes of People Attended by a Portuguese Community-Based Association: A Thematic Analysis. *Healthcare.* 10(10):1819. https://doi.org/10.3390/healthcare10101819

**Disclosure of Interest:**

None Declared

